# Continuum-Scale Modeling of Liquid Redistribution in a Stack of Thin Hydrophilic Fibrous Layers

**DOI:** 10.1007/s11242-018-0999-0

**Published:** 2018-01-12

**Authors:** Amir Hossein Tavangarrad, Behzad Mohebbi, S. Majid Hassanizadeh, Rodrigo Rosati, Jan Claussen, Bernhard Blümich

**Affiliations:** 1 Department of Earth Sciences, Environmental Hydrogeology Group, Utrecht University, Princetonplein 9, 3584 CC Utrecht, The Netherlands; 2 Procter & Gamble Service GmbH, Sulzbacher Str. 40, 65824 Schwalbach am Taunus, Germany; 3 Institut für Technische und Makromolekulare Chemie, RWTH Aachen, 52074 Aachen, Germany

**Keywords:** Thin porous media, Moisture redistribution, Low-field NMR, Fibrous layer, Dynamic capillary pressure, Reduced continua model

## Abstract

Macroscale three-dimensional modeling of fluid flow in a thin porous layer under unsaturated conditions is a challenging task. One major issue is that such layers do not satisfy the representative elementary volume length-scale requirement. Recently, a new approach, called reduced continua model (RCM), has been developed to describe multiphase fluid flow in a stack of thin porous layers. In that approach, flow equations are formulated in terms of thickness-averaged variables and properties. In this work, we have performed a set of experiments, where a wet $260\hbox {-}\upmu \hbox {m}$-thin porous layer was placed on top of a dry layer of the same material. We measured the change of average saturation with time using a single-sided low-field nuclear magnetic resonance device known as NMR-MOUSE. We have employed both RCM and the traditional Richards equation-based models to simulate our experimental results. We found that the traditional unsaturated flow model cannot simulate experimental results satisfactorily. Very close agreement was obtained by including the dynamic capillary term as postulated by Hassanizadeh and Gray in the traditional equations. The reduced continua model was found to be in good agreement with the experimental result without adding dynamic capillarity term. Moreover, the computational effort needed for RCM simulations was one order of magnitude less than that of traditional models.

## Introduction

Thin nonwoven fibrous materials have a wide range of applications in different industries and consumer products. Examples are papers, filters, insulators, fuel cells, fluid absorbent or barrier materials, textiles, diapers, and hygienic pads. An important process of interest is the flow of liquids through a stack of thin porous layers under unsaturated conditions. There are various research questions in the field of thin porous media related to identifying theoretical, numerical, and experimental techniques (see, for example, Prat and Agaësse 2015; Aslannejad and Hassanizadeh 2017).

Thin porous layers behave differently from thicker porous media. Some physical phenomena whose impact is weak or even negligible in an ordinary porous medium can become dominant in a thin medium (Prat and Agaësse 2015). Ceballos et al. (2011) defined a thin porous layer based on the number of pores along the thickness dimension. A discrete representation of the porous layer as a network of interconnected capillaries was used in their work to simulate a pore-scale drainage process in a thin fibrous layer. They showed that number of variables in their thin porous medium, such as the invading phase overall saturation at the end of the displacement and the receding phase saturation profiles, are different from those of a thick one for a particular displacement process.

Research on understanding the behavior of thin porous media under unsaturated flow conditions has been done on both continuum and pore scales. Rebai and Prat (2009) used traditional continuum models and a pore network model to predict the water distribution across a thin layer. They stated that traditional continuum-scale modeling cannot be relied on to compute the water distribution along the thickness of thin layers because the pore size is comparable to the thickness. Although the applicability of traditional continuum models, like the Richards equation (1931), to thin fibrous media is questionable, they are commonly used to predict water infiltration (Landeryou et al. 2005; Tafreshi and Bucher 2013; Jaganathan et al. 2009; Landeryou et al. 2003) or drainage (Ashari et al. 2011; Ashari and Tafreshi 2009).

Landeryou et al. (2005) studied in-plane infiltration in horizontal and inclined thin fibrous sheets experimentally and numerically. Results of their continuum Richards modeling for a horizontal sheet, subject to a capillary-driven line-source boundary condition, showed that measured saturation profiles were significantly sharper than modeling results. For the inclined sheet experiments, their numerical results for a point-source boundary condition overpredicted the rate of water infiltration in cross-slope direction, while satisfactory results were obtained for the down-slope direction. They proposed that by including a percolation threshold in relative permeability function, one may be able to reduce calculated infiltration rate in lateral direction.

Tafreshi and Bucher (2013) also studied the in-plane absorption of water in a thin fibrous sheet. They showed that simulation results of Richards equation can be verified against an analytical expression given by Marmur (1988). They also reviewed in-plane infiltration experimental studies in the literature that were compared with Marmur equation. They also stated that water infiltrates a thin fibrous sheet preferably in the direction of fibers. Consequently, if fibers are predominantly perpendicular to the direction of flow, their impeding effects should be expressed in relative permeability function.

Among others, Ashari et al. (2011) used Richards equation to model drainage of fluid from thin nonwoven fibrous layer in the through-plane direction. They pulled around a partially saturated thin layer over a rough solid surface causing water to leave the layer. They measured average saturation of the layer as a function of time. They used a special boundary condition containing a coefficient which was fitted in order to match measured data.

Qin and Hassanizadeh (2015) proposed two definitions for a thin porous layer: physically thin layer and geometrically thin layer. A ‘physically thin layer’ is a layer that does not satisfy the representative elementary volume (REV) length-scale requirement. According to that requirement, macroscale properties should be defined for an averaging volume (REV) whose size is more than one order of magnitude larger than pore sizes and more than one order of magnitude smaller than overall size of porous material. This means that for macroscale (continuum-scale) equations to be valid, the material thickness must be a few orders of magnitude larger than pore or grain sizes. Therefore, a layer whose thickness is only 10 times larger than its mean pore size does not meet REV requirements. So, they argued that the applicability of continuum models to physically thin porous media is questionable. A ‘geometrically thin porous layer’ was defined as a layer that satisfies the REV requirement, but its thickness is much smaller than its planar dimensions. Recently, Qin and Hassanizadeh (2014) developed a new approach for modeling multiphase flow in a stack of physically thin porous layers, based on thermodynamic considerations. They referred to it as the reduced continua model (RCM). All governing equations in this model are formulated in terms of thickness-averaged properties. Therefore, a 3D system is reduced to a two-dimensional domain, which needs to be discretized only in the lateral directions. Qin and Hassanizadeh (2015) found good agreement between traditional continuum-scale models and the RCM for modeling the fluid flow in a thin hydrophobic material. However, their results have not yet been validated against any experimental data.

Experimental techniques for the investigation of fluid distributions in thin layers and their dynamics are challenging. Commonly, gravimetric measurements are used to quantify the amount of liquid in layered textile materials. However, these methods cannot reveal details of the liquid distribution or flow dynamics inside the material.

There are several nondestructive techniques, such as X-ray tomography (Weder et al. 2006) and scanning neutron radiology (Weder et al. 2004; Pel et al. 1993), that have been employed to investigate the movement and distribution of water within porous substrates. However, using these instruments in bench-top setups is difficult and special precautions are required to obtain meaningful information. Magnetic resonance imaging (MRI) is another method that enables noninvasive monitoring of fluid flow through porous materials (Leisen et al. 1998). However, MRI has been used mainly for thicker-layered materials, such as carpets, to study the evaporation and drying of substrates (Leisen and Beckham 2001; Carr et al. 1998). Alternatively, nuclear magnetic resonance (NMR) has been used for determining liquid distributions inside porous materials, such as soil, concrete, building materials, and food (Blümich et al. 2009, 2011).

**Table 1:** Properties of fibrous layer used in this study

Parameter	Value	Unit
Porosity (${\varphi }$)	0.9	–
Fiber radius (*r*)	10	$\upmu \hbox {m}$
Fiber density ($\rho ^{\mathrm{f}}$)	0.92	$\hbox {gr}/\hbox {cm}^{3}$
Permeability (*K*)	$2.05\times 10^{-10}$	$\hbox {m}^{2}$
Fitting parameters in the VG model of imbibition curve ($\alpha$)	0.00378	$1/\hbox {Pa}$
Fitting parameters in the VG model of imbibition curve (*n*)	8.53	–
Irreducible saturation ($S_{\mathrm{ir}}$)	0.02	–
Fitting parameters in Durner model of drainage curve ($w_{\mathrm{f}} ,w_{\mathrm{m}}$)	0.42, 0.58	–
Fitting parameters in Durner model of drainage curve ($\alpha _{\mathrm{f}}, \alpha _{\mathrm{m}}$)	0.0026, 0.00056	$1/\hbox {Pa}$
Fitting parameters in Durner model of drainage curve ($n_{\mathrm{f}}, n_{\mathrm{m}}$)	2.07, 6.64	–

**Fig. 1:**
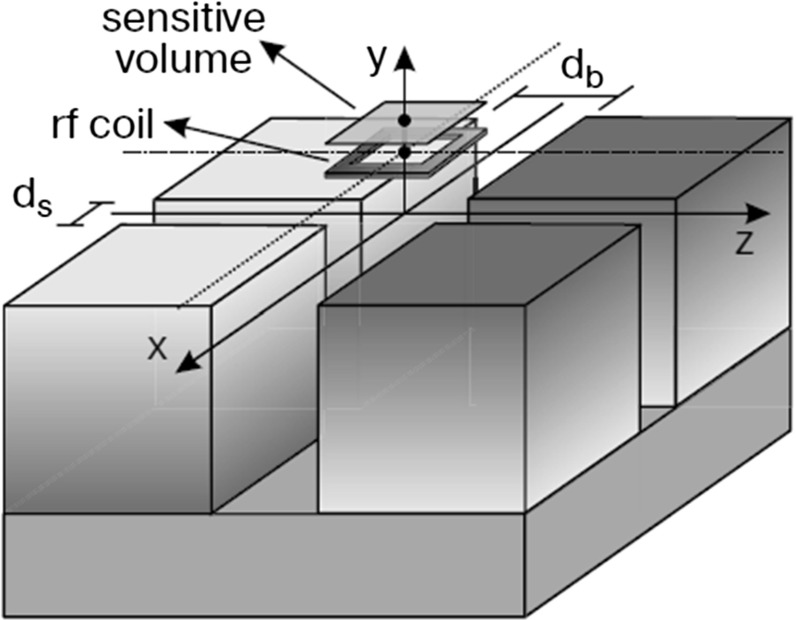
Magnet arrangement in a NMR-MOUSE which collects signal from a flat sensitive volume above the sensor. The direction of the polarization of the magnets is indicated by the gray scale

In this study, a bench-top single-sided NMR device called NMR-MOUSE (**Mo**bile **U**niversal **S**urface **E**xplorer) (Perlo et al. 2005) has been used for dynamic measurements of drainage processes through thin porous layers (Fig. 1). In particular, the change of water saturation in a wet layer draining to an underlying dry layer is monitored. We have also performed continuum-scale modeling of liquid flow from the top to the bottom layer, employing both traditional Richards equation and the new RCM approach. The two models were calibrated in order to simulate drainage of a hydrophilic thin fibrous layer. This is the first time that the RCM approach has been compared to experimental results. In addition, in both models, we have accounted for a dynamic capillarity term as postulated by Hassanizadeh and Gray (1993).

## Experimental Methods and Material

### Materials

The material used in this study was a thin nonwoven porous layer made of polyolefin-based fibers. It is thermally bonded in some regions during the fabrication process with a lozenge bonding pattern. A specific surfactant has been added to this material to make it hydrophilic. The layer was about $260 \, \upmu \hbox {m}$ thick with around 90% porosity. Other properties including fiber radius and density are given in Table 1. A saline solution, made of 0.9% NaCl (by weight) and distilled water, was chosen as the working liquid. The solution has a surface tension of $72.5 \, \hbox {mN}/\hbox {m}$, a density of $1.005 \, \hbox {g}/\hbox {cm}^{3}$, and a viscosity of $1.019 \, \hbox {mPa}\,\hbox {s}$. The fibrous material exhibits negligible swelling in such a solution.

### NMR Instrument

The NMR-MOUSE (Magritek Europe, Aachen, Germany) consists of a surface rf (radio frequency) coil placed above four permanent magnets (Fig. 1). Two magnets with the same polarization were separated by a distance ${d}_{\mathrm{s}}$. The magnets with opposite polarization were separated by a distance ${d}_{\mathrm{b}}$, which was larger than ${d}_{\mathrm{s}}$. The NMR-MOUSE used in this work generated a uniform magnetic field gradient of 8 T/m in a magnetic field of 0.32 T ($^{1}\hbox {H}$ frequency of 13.8 MHz) in a sensitive volume located at 25 mm above the surface of magnets (see Fig. 1). The sensitive volume had a thickness of $200 \, \upmu \hbox {m}$ and lateral dimension of 40 mm by 40 mm. The elevation of sensitive volume could be varied with the help of a high-precision lift moving the magnets up and down, while keeping the sample fixed. The NMR signal was acquired with a CPMG multi-echo sequence (Carr and Purcell 1954).

A quadratic 40-mm-diameter surface coil was used to excite the NMR signal from the sensitive volume. The sensitive volume thickness $\Delta z$ is proportional to the bandwidth of the rf pulse according to the following formula:

1 $$\begin{aligned} \Delta z=2 \uppi \Delta \nu /G_z \, \upgamma , \end{aligned}$$

where $\Delta \nu$ is the excitation bandwidth, $G_z$ is the magnetic field gradient, and $\upgamma$ is the gyromagnetic ratio. In the CPMG sequence, the spatial resolution and the thickness of the sensitive volume could be adjusted by changing the number (*m*) of acquisition points in each echo and the dwell time (*dw*); this resulted in an acquisition time of $m\cdot dw$ for each echo. A $200\hbox {-}\upmu \hbox {m}$-thick sensitive volume was created in our experiments. With each CPMG sequence, 128 echoes were acquired (echo time ${t}_{\mathrm{E}} =90 \, \upmu \hbox {s}$, repetition time ${t}_{\mathrm{R}} =200 \, \hbox {ms}$, number of scans = 1500).

### Water Redistribution Experiment

**Fig. 2:**
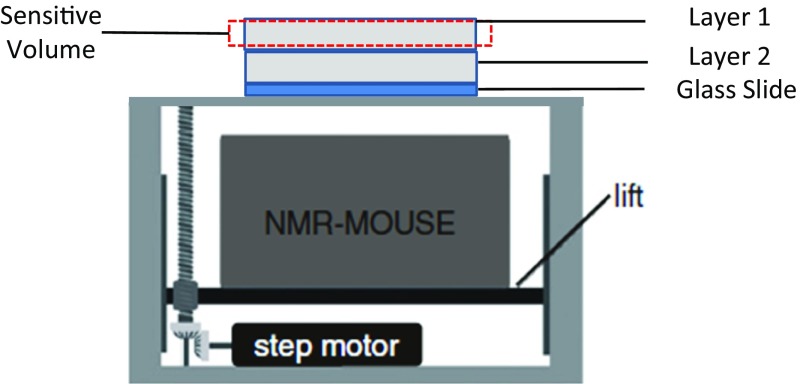
Placement of two thin nonwoven layers on top of the NMR sensor where layer 1 is fully saturated and layer 2 is dry

The main objective of this experiment was to monitor the dynamic drainage process of a thin porous material. This was done by placing a fully saturated layer of nonwoven fabric on top of a dry layer and monitoring the change of saturation in the wet layer (Fig. 2). Experiments were performed in a climate-controlled environment, at $24\,^{\circ }\hbox {C}$ and a humidity of 46%.

For the measurements, two identical samples with dimension of $40 \times 40 \, {\hbox {mm}}^{2}$ and thickness of $260 \, \upmu \hbox {m}$ were cut. A glass slide with thickness of $150 \, \upmu \hbox {m}$ was placed on top of the coil, and a dry sample was placed on it. Then, the other sample was saturated with the saline solution and placed over the dry sample. Next, using the high-precision lift, the sensitive volume with a thickness of $200 \, \upmu \hbox {m}$ was positioned inside the upper layer.

In order to account for the evaporation from the wet layer, an additional experiment was done in the same climate-controlled laboratory. In this experiment, a fully saturated layer was placed on a balance and the weight loss over time was measured. This allowed us to compute the evaporation rate. The experimental results for the drainage were corrected by taking the measured evaporation rate into an account.

## Modeling Approaches

### Richards Model

Here, we describe two approaches for modeling liquid flow through a stack of thin layers. Most commonly used models for flow of a liquid through a thin porous medium under unsaturated conditions are based on the Richards equation, which assumes that the air phase is infinitely mobile. It is obtained by writing the mass conservation of the wetting phase in following form:

2 $$\begin{aligned} \frac{\partial (\rho ^{\mathrm{w}}{\varphi }S^{\mathrm{w}})}{\partial t}+{\varvec{\nabla }}. ( {\rho ^{\mathrm{w}}{\varvec{v}}^{\mathrm{w}}})=0, \end{aligned}$$

where $\rho ^{\mathrm{w}}$ is the mass density of wetting phase, ${\varphi }$ is the porosity, and $S^{\mathrm{w}}$ is the saturation of the wetting phase. When neglecting gravity the Darcy velocity vector ${\varvec{v}}^{\mathrm{w}}$ is given by:

3 $$\begin{aligned} {\varvec{v}}^{\mathrm{w}}=-\frac{k^{\mathrm{r}}K}{\mu ^{\mathrm{w}}}{\varvec{\nabla }} P^{\mathrm{w}}, \end{aligned}$$

where *K* is the intrinsic permeability, $k^{\mathrm{r}}$ is the relative permeability of the wetting phase, $\mu ^{\mathrm{w}}$ is the dynamic viscosity of the wetting phase, and ${\varvec{\nabla }} P^{\mathrm{w}}$ is the pressure gradient. In our study, gravity is negligible due to the small thickness of the layers.

To finalize the relationships between water pressure, saturation, and relative permeability, the macroscopic capillary pressure is commonly assumed to be equal to the difference in fluid pressures:

4 $$\begin{aligned} P^{\mathrm{a}}-P^{\mathrm{w}}=P^{\mathrm{c}} ({S^{\mathrm{w}}}), \end{aligned}$$

where $P^{\mathrm{a}}$ is the air pressure and $P^{\mathrm{c}} ({S^{\mathrm{w}}})$ is the capillary pressure, which traditionally is assumed to be solely a function of saturation. However, based on a rigorous thermodynamic approach, Hassanizadeh and Gray (1990) proposed the existence of a dynamic component that depends on $\frac{\partial S^{\mathrm{w}}}{\partial t}$. Later on, Hassanizadeh and Gray (1993) developed the following linear approximation for the difference in fluid pressures under dynamic conditions:

5 $$\begin{aligned} P^{\mathrm{a}}-P^{\mathrm{w}}= P^{\mathrm{c}} ({S^{\mathrm{w}}}) - \tau \frac{\partial S^{\mathrm{w}}}{\partial t}, \end{aligned}$$

where $\tau$ is a material coefficient, which should be determined experimentally. Reviews of issues related to dynamic capillarity effect can be found in Hassanizadeh et al. (2002) and Joekar-Niasar and Hassanizadeh (2012).

A nonlinear relationship suggested by Van Genuchten (VG) can be used for the capillary pressure function as follows:

6 $$\begin{aligned} P^{\mathrm{c}} ({S_{\mathrm{e}}}) = \frac{1}{\alpha } \left( {S_{\mathrm{e}}^{-1/m}-1}\right) ^{1/n}, \end{aligned}$$

where $\alpha$ and *n* are fitting parameters, $m=1-1/n$, and $S_e = ({S^{\mathrm{w}}-S_{\mathrm{ir}}})/ ({1-S_{\mathrm{ir}}})$ in which $S_{\mathrm{ir}}$ is the irreducible water saturation. Due to very large porosity of the samples, no residual air needs to be considered. In some cases, a modified VG equation needs to be used, where *m* is also considered as a fitting parameter. All parameters in this equation have to be determined experimentally.

Furthermore, the relative permeability is assumed to be a function of saturation. One common formula for relative permeability was suggested by Genuchten (1980) as:

7 $$\begin{aligned} k^{\mathrm{r}} ({S^{\mathrm{w}}}) = S_{\mathrm{e}}^l \left( {1- \left( {1-S_{\mathrm{e}}^{1/m}}\right) ^{m}}\right) ^{2}, \end{aligned}$$

where *m* is the same parameter that appears in the $P^{\mathrm{c}}-S^{\mathrm{w}}$ relationship and $l=0.5$.

Another relationship is a power-law model based on the empirical correlation of Brooks and Corey (1964):

8 $$\begin{aligned} k^{\mathrm{r}} ({S^{\mathrm{w}}}) = (S^{\mathrm{w}})^{\delta }, \end{aligned}$$

where $\delta$ is a material property and should be determined experimentally. There exist some experimental studies for the determination of $\delta$ for a thin fibrous layer (Sole 2008; Hussaini and Wang 2010). For in-plane permeability, Hussaini and Wang (2010) found a value of $\delta =4$ for a thin nonwoven fibrous layer, but they report large uncertainties in their measurements (Hussaini and Wang 2010). A wide range of values for this coefficient ($3\le \delta \le 6$) has been used in numerical simulations of thin fibrous layers (Jaganathan et al. 2009; Landeryou et al. 2005; Ashari and Tafreshi 2009).

For the case of a heterogeneous pore structure, a multimodal function proposed by Durner (1994) can be used to define the hydraulic functions. For instance, in the case of a bimodal retention function, Eqs. (6) and (7) are replaced by the following two equations (Durner 1994; Genuchten and Pachepsky 2011):

9 $$\begin{aligned}&\displaystyle S_{\mathrm{e}} (h) = \frac{w_{\mathrm{f}} }{[{1+ |{\alpha _{\mathrm{f}} h}|^{n_{\mathrm{f}}}}]^{m_{\mathrm{f}} }}+\frac{w_{\mathrm{m}} }{\left[ {1+ |{\alpha _{\mathrm{m}} h}|^{n_{\mathrm{m}} }}\right] ^{m_{\mathrm{m}} }}, \end{aligned}$$

10 $$\begin{aligned}&\displaystyle k^{\mathrm{r}} ({S_\mathrm{e}}) = \frac{(w_{\mathrm{f}} S_{{\mathrm{e}}_{\mathrm{f}}} +w_{\mathrm{m}} S_{{\mathrm{e}}_{\mathrm{m}}})^{l_{\mathrm{d}}} \, \left\{ {w_{\mathrm{f}} \alpha _{\mathrm{f}} \left( {1-\left( {1-S_{\mathrm{e}_{\mathrm{f}} }^{1/m_{\mathrm{f}} } } \right) ^{m_{\mathrm{f}} }} \right) +w_{\mathrm{m}} \alpha _{\mathrm{m}} \left( {1-\left( {1-S_{{\mathrm{e}_{\mathrm{m}}}}^{1/m_{\mathrm{m}} } } \right) ^{m_{\mathrm{m}} }} \right) } \right\} ^{2}}{({w_{\mathrm{f}} \alpha _{\mathrm{f}} +w_{\mathrm{m}} \alpha _{\mathrm{m}}})^{2}},\nonumber \\ \end{aligned}$$

where *h* is the capillary pressure head, subscripts $\hbox {f}$ and $\hbox {m}$ refer to fracture (macropore) and matrix (micropore), respectively, $\alpha _{\mathrm{f}}$, $n_{\mathrm{f}},$, $\alpha _{\mathrm{m}}$, $n_{\mathrm{m}}$ are fitting parameters, and $w_{\mathrm{f}}$ and $w_{\mathrm{m}}$ are weight factors for overlapping regions ($w_{\mathrm{f}} +w_{\mathrm{m}} =1$). Further, $l_{\mathrm{d}} =0.5$, $m_{\mathrm{f}} =1-\frac{1}{n_{\mathrm{f}}}$, $m_{\mathrm{m}} =1-\frac{1}{n_{\mathrm{m}}}$, and $S_{\mathrm{e}} =w_{\mathrm{f}} S_{{\mathrm{e}}_{\mathrm{f}}} +w_{\mathrm{m}} S_{{\mathrm{e}}_{\mathrm{m}}}$.

### Reduced Continua Model (RCM)

Application of Richards equation requires discretization of the modeling domain over the layer thickness. An alternative model is the reduced continua model (RCM), where equations of water flow are formulated in terms of thickness-averaged material properties. Therefore, as mentioned before, the dimensionality of a problem is reduced by one order and needs to be discretized in planar directions only. In this approach, macroscale balance laws are formulated based on measurable thickness-averaged properties. The layer–layer interactions are accounted for by a transfer term for mass, heat, and momentum. Closed from of governing equations are derived based on the second law of thermodynamics and linearization theory. The mass conservation for liquid transport in a layer *i* can be written as (Qin and Hassanizadeh 2015):

11 $$\begin{aligned} {\left( {\frac{\partial ({b\rho ^{\mathrm{w}}{\varphi }S^{\mathrm{w}}})}{\partial t}+{\varvec{\nabla }}_{\mathrm{h}} .\left( {b\rho ^{\mathrm{w}} {\varvec{v}}_{\mathrm{h}}^{\mathrm{w}} } \right) } \right) } \Big |_i = {Q_{\mathrm{T}}^{\mathrm{w}} } \big |_i + {Q_{\mathrm{B}}^{\mathrm{w}} } \big |_i , \end{aligned}$$

where ${\varvec{v}}_{\mathrm{h}}^{\mathrm{w}}$ denotes the planar Darcy velocity vector, ${\varvec{\nabla }}_{\mathrm{h}}$ is the planar spatial gradient, ${Q_{\mathrm{T}}^{\mathrm{w}}} \big |_i$ and ${Q_{\mathrm{B}}^{\mathrm{w}}}\big |_i$ indicate liquid inflow and outflow from the layer *i*, respectively, and *b* is the layer thickness.

The planar velocity ${\varvec{v}}_{\mathrm{h}}^{\mathrm{w}}$ is given by a Darcy-like equation, which in the case of negligible gravity reads:

12 $$\begin{aligned} {{\varvec{v}}_{\mathrm{h}}^{\mathrm{w}}} \big |_i = {\left( {-\frac{k^{\mathrm{r}}K}{\mu ^{\mathrm{w}}} {\varvec{\nabla }}_{\mathrm{h}} P^{\mathrm{w}}} \right) } \Big |_i, \end{aligned}$$

where $P_i^w$ is the thickness-averaged wetting phase pressure.

**Fig. 3:**
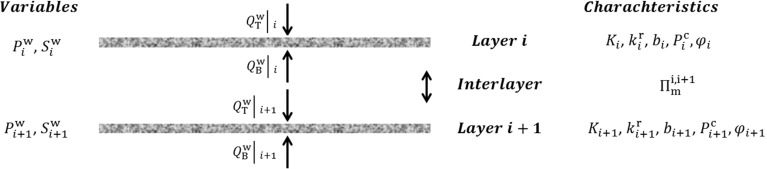
A sketch of stack of two layers with their characteristics and variables used in RCM model

One major issue in the RCM is the characterization of the terms ${Q_{\mathrm{T}}^{\mathrm{w}}} \big |_i$ and ${Q_{\mathrm{B}}^{\mathrm{w}}} \big |_i$. Based on thermodynamic considerations and some simplifying assumptions, Qin and Hassanizadeh (2014) proposed the following equation:

13 $$\begin{aligned} {Q_{\mathrm{B}}^{\mathrm{w}}} \big |_i =- {Q_{\mathrm{T}}^{\mathrm{w}}} \big |_{i+1} = {\Pi }_{\mathrm{m}}^{i,i+1} \left( {P_{i+1}^{\mathrm{w}} -P_i^{\mathrm{w}}}\right) , \end{aligned}$$

where the subscripts *i* and $i+1$ denote two neighboring layers, ${Q_{\mathrm{B}}^{\mathrm{w}}}|_i$ is the flux leaving the bottom of top layer, while ${Q_{\mathrm{T}}^{\mathrm{w}}}|_{i+1}$ is the water flux entering the upper surface of the bottom layer, and $\Pi _m^{i,i+1}$ is a mass transfer coefficient (Fig. 3). The coefficient $\Pi _m^{i,i+1}$ is specific to the two layers, but also includes the nature of contact surface between the two layers. For example, pressing the two layers together may change the value of this mass transfer coefficient. Assuming continuity of the flow rate at the contact area, the following equation can be obtained for the mass transfer coefficient:

14 $$\begin{aligned} \prod \limits _{{m}}^{{i}, {i}+1} =\frac{2}{b_i +b_{i+1}} \rho ^{\mathrm{w}} \frac{{\overline{K_{\mathrm{t}}}}}{\mu ^{\mathrm{w}}}, \end{aligned}$$

where ${\overline{K_{\mathrm{t}}}}$ is an effective through-plane permeability of the two layers and is defined as follows:

15 $$\begin{aligned} {\overline{K_{\mathrm{t}}}} =\left( {\frac{b_i +b_{i+1} }{b_{i+1} K_i k_i^{\mathrm{r}} +b_i K_{i+1} k_{i+1}^{\mathrm{r}} }K_i k_i^{\mathrm{r}} K_{i+1} k_{i+1}^{\mathrm{r}} } \right) , \end{aligned}$$

Equation (15) is analogous to Kirchhoff’s law for electrical resistors. Note that ${\overline{K_{\mathrm{t}}}}$ is a function of saturations of the two layers through their relative permeability functions $k_i^{\mathrm{r}}$ and $k_{i+1}^{\mathrm{r}}$. Alternatively, the effective through-plane permeability ${\overline{K_{\mathrm{t}}}}$ can be written in the following form, as proposed by Qin and Hassanizadeh (2015):

16 $$\begin{aligned} {\overline{K_{\mathrm{t}}}} =\frac{b_i +b_{i+1} }{b_{i+1} K_i +b_i K_{i+1} }K_i K_{i+1} f\left( {S_i^{\mathrm{w}}, S_{i+1}^{\mathrm{w}}} \right) . \end{aligned}$$

**Fig. 4:**
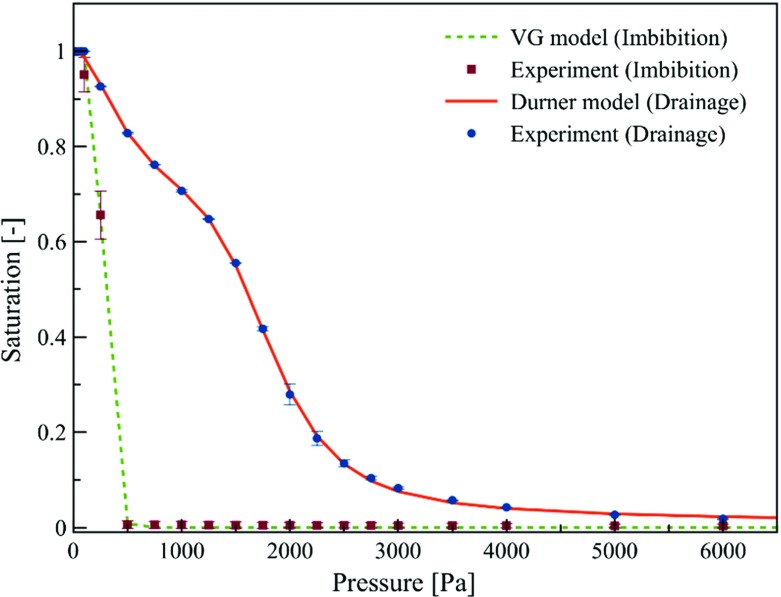
Measured capillary pressure–saturation data points for a single layer of fabric. The error bars are based on duplicate measurements. The imbibition data were fitted with the Van Genuchten formula and the drainage data with the Durner formula

Here, the function $f ({S_i^\mathrm{w}, S_{i+1}^\mathrm{w}})$ accounts for the role of relative permeability functions of the two layers. Qin and Hassanizadeh (2015) proposed the following equation for this function:

17 $$\begin{aligned} f\left( {S_i^{\mathrm{w}}, S_{i+1}^{\mathrm{w}}} \right) = \frac{1}{2}\left( {\left( S_i^{\mathrm{w}} \right) ^{\mathrm{\gamma }_i} + \left( S_{i+1}^{\mathrm{w}} \right) ^{\mathrm{\gamma }_{i+1} }} \right) , \end{aligned}$$

where ${\upgamma }_i$ and ${\upgamma }_{i+1}$ are to be determined experimentally. In addition to Eqs. (11)–(15), equations for capillary pressure and relative permeability are given by (6)–(7) or (9)–(10). In our study, Eq. (6) was used to fit measured imbibition data points, whereas Eq. (9) was found to fit measured drainage data points more closely (Fig. 4).

## Numerical Simulations

### Values of Model Parameters

Properties of the layer, including porosity and permeability, are listed in Table 1. It should be noted that porosity in Table 1 was measured using a Textile Research Institute Autoporosimeter (Miller and Tyomkin 1994), and the fiber radius was estimated from CT images of the layer.

The intrinsic permeability of the fibrous layer was measured in the laboratory with the falling head method (Head 1992). However, because the initial head of 15 cm used in the measurements could have changed the layer porosity, we also used a number of empirical equations for the calculation of the permeability as a function of porosity and fiber radius (Tomadakis and Robertson 2005; Jackson and James 1986; Davies 1973; Spielman and Goren 1968). For example, Jackson and James (1986) developed the following formula based on flow through three-dimensional arrays of cylinders to predict the permeability of a highly porous fabric:

18 $$\begin{aligned} K= \frac{3 \, r^{2}}{20 ({1-{\varphi }})} ({-\ln ({1- {\varphi }})-0.931}), \end{aligned}$$

where *r* is the fiber radius and ${\varphi }$ the porosity of the medium. All formulas gave almost the same value of the intrinsic permeability, which was comparable to the measured value. The value reported in Table 1 was obtained from the formula of Jackson and James (1986).

A Pore Volume Distribution Autoporosimeter (PVD-Autoporosimeter), from the Textile Research Institute, called TRI-PVD (Miller and Tyomkin 1994), was used to measure capillary pressure–saturation data points from multistep inflow/outflow measurements. Measurements were taken for a circular piece of sample with the diameter of 50 mm. Results are shown in Fig. 4. The best fit to the drainage data points was obtained with the Durner bimodal formula (9), whereas the Van Genuchten formula (6) was found to give the best fit for the imbibition data points (Fig. 4). The corresponding fitted parameters are given in Table 1. These parameters were also used in the corresponding relative permeability functions given by Eqs. (10) and (7).

### Richards Model Solution

The governing equations were solved using COMSOL (COMSOL, Burlington, USA), which is a finite element commercial software. For the simulation of the redistribution experiment, the modeling domain consisted of two layers of fabric with the total thickness of $526 \, \upmu \hbox {m}$. Because the layers were homogenous, the upper layer was fully and uniformly saturated, and the bottom layer was dry, we only needed to solve a one-dimensional downward flow problem. Our preliminary simulations using 1D Richards equation showed that 526 elements along the thickness provided mesh-independent results. Equations (2)–(3) and (5) were solved for both layers in this approach.

A no-flux boundary condition was imposed at both the top and the bottom boundaries of the modeling domain. At the interface between the two layers, continuity of both pressure and flux was assumed for the Richards model.

### RCM Solution

For the RCM, the one-dimensional simulation domain reduces to only two grid points, representing the wet and dry layers. Thus, governing equations for the RCM approach reduce to an ordinary differential equation. Given the fact that we have a close system, the sum of average saturation of two layers is constant and equal to unity all times:

19 $$\begin{aligned} S_1^{\mathrm{w}} + S_2^{\mathrm{w}} =1, \end{aligned}$$

where $S_1^{\mathrm{w}}$ and $S_2^{\mathrm{w}}$ indicate water saturation of top and bottom layers, respectively. Also, Eq. (13) for mass transfer term of the wet layer can be rewritten as:

20 $$\begin{aligned} Q_{\mathrm{B}1}^{\mathrm{w}} = {\Pi }_{\mathrm{m}}^{1,2} \left( {P_2^{\mathrm{w}} -P_1^{\mathrm{w}} } \right) = {\Pi }_{\mathrm{m}}^{1,2} \left[ {-P_{\mathrm{imb}}^{\mathrm{c}} \left( {S_2^{\mathrm{w}} } \right) +P_{\mathrm{dra}}^{\mathrm{c}} \left( {S_1^{\mathrm{w}} } \right) } \right] , \end{aligned}$$

where we make use of the fact that air pressure is constant. As explained in Section 3.2, we can use two different formulations for the mass transfer coefficient ${\Pi }_{\mathrm{m}}^{1,2}$. Given the fact that two layers were identical, Eqs. (14) and (15) for the first formulation reduce to:

21 $$\begin{aligned} \Pi _{\mathrm{m}1}^{1,2} =\frac{2}{b} \frac{\rho ^{\mathrm{w}}}{\mu ^{\mathrm{w}}}\frac{Kk_1^{\mathrm{r}} k_2^{\mathrm{r}} }{k_1^{\mathrm{r}} +k_2^{\mathrm{r}}}. \end{aligned}$$

For the second formulation, the simplified form of Eq. (17) proposed by Qin and Hassanizadeh (2015) was used and mass transfer coefficient was described by combining Eqs. (14), (16), and (17):

22 $$\begin{aligned} \Pi _{\mathrm{m}2}^{1,2} =\frac{2}{b} \frac{\rho ^{\mathrm{w}}}{\mu ^{\mathrm{w}}} K \frac{\left( S_1^{\mathrm{w}} \right) ^{\delta }+\left( S_2^{\mathrm{w}}\right) ^{\delta }}{2}, \end{aligned}$$

where $\delta$ is the same parameter as in the relative permeability formula (6). Since no measured value of $\delta$ for our thin fabric layer was available, this parameter was set equal to 4 based on suggestion of Hussaini and Wang (2010).

Based on the first formulation of mass transfer coefficient $\Pi _{\mathrm{m}1}^{1,2}$, the following equation should be solved for the top layer, by combining Eqs. (11), (19), (20), and (21):

23 $$\begin{aligned} \frac{dS_1^{\mathrm{w}} }{dt}=\frac{2K}{b^{2}\mu ^{\mathrm{w}}{\varphi }_1} \frac{k_1^{\mathrm{r}} \left( {S_1^{\mathrm{w}} } \right) k_2^{\mathrm{r}} \left( {1-S_1^{\mathrm{w}} } \right) }{k_1^{\mathrm{r}} \left( {S_1^{\mathrm{w}} } \right) +k_2^{\mathrm{r}} \left( {1-S_1^{\mathrm{w}} } \right) } \left[ {-P_{\mathrm{imb}}^{\mathrm{c}} \left( {1-S_1^{\mathrm{w}} } \right) +P_{\mathrm{dra}}^{\mathrm{c}} \left( {S_1^{\mathrm{w}} } \right) } \right] . \end{aligned}$$

Note that $P_{\mathrm{dra}}^{\mathrm{c}} ({S_1^{\mathrm{w}}})$ is given by Durner formula and VG model was used for $P_{\mathrm{imb}}^{\mathrm{c}} ({1-S_1^{\mathrm{w}}})$ function. Relative permeability functions were determined based on fitted parameters accordingly.

Based on the second formulation for the mass transfer term, the following equation should be solved for the top layer based on Eqs. (11), (19), (20), and (22):

24 $$\begin{aligned} \frac{dS_1^{\mathrm{w}} }{dt}=\frac{2K}{b^{2}\mu ^{\mathrm{w}}{\varphi }_1} \frac{\left( S_1^{\mathrm{w}}\right) ^{4}+ \left( 1-S_1^{\mathrm{w}} \right) ^{4}}{2}\left[ {-P_{\mathrm{imb}}^{\mathrm{c}} \left( {1-S_1^{\mathrm{w}} } \right) +P_{\mathrm{dra}}^{\mathrm{c}} \left( {S_1^{\mathrm{w}} } \right) } \right] . \end{aligned}$$

The third set of simulations with RCM approach was performed, where we used Eq. (5) instead of Eq. (4) in order to include dynamic capillarity given by the following equation:

25 $$\begin{aligned} \frac{dS_1^{\mathrm{w}} }{dt}=\frac{2K}{b^{2}\mu ^{\mathrm{w}}{\varphi }_1} \frac{\left( S_1^{\mathrm{w}}\right) ^{4}+ \left( 1-S_1^{\mathrm{w}} \right) ^{4}}{2}\left[ {-P_{\mathrm{imb}}^{\mathrm{c}} \left( {1-S_1^{\mathrm{w}} } \right) +P_{\mathrm{dra}}^{\mathrm{c}} \left( {S_1^{\mathrm{w}} } \right) } - 2\tau {\frac{{dS}_{1}^w}{dt}}\right] .\quad \end{aligned}$$

Despite the fact that we have simple ordinary differential equation, due to the strong nonlinearity of hydraulic functions, it was not possible to solve them analytically. Therefore, solutions of Eqs. (23), (24), and (25) were obtained numerically using COMSOL software.

In the following section, the results for the two different modeling approaches are compared to experimental data generated by the NMR-MOUSE.

## Results

### Water Redistribution Experiment

**Fig. 5:**
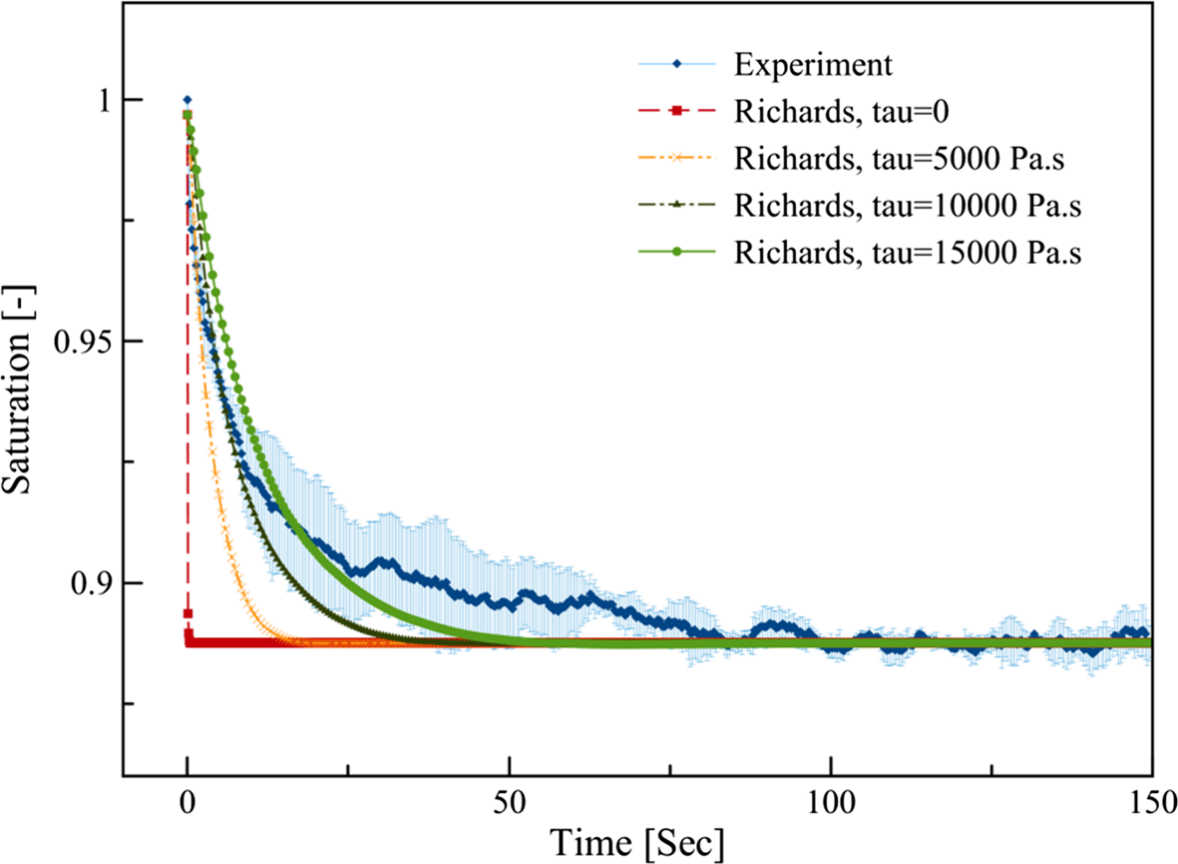
Temporal evolution of the average saturation of the top layer obtained from solving the Richards equation in comparison with the experimental NMR data. Shaded area represents standard deviation of three replicates

The temporal evolution of the corrected water saturation, as measured by the NMR-MOUSE for a $200\hbox {-} \upmu \hbox {m}$-thick zone within the wet layer is shown by blue symbols in Fig. 5. The raw data of experimental results were corrected based on measured evaporation rate. The light blue shadow lines represent standard deviations of three replicates. As it can be seen, the saturation of top layer decreases rapidly and reaches an equilibrium value within about 3 min. Saturation change was quite small, reaching an equilibrium value of 88%. This means that the dry layer reached on average saturation of 12% only. The fact that a full redistribution does not occur when wet and dry layers are brought into contact is well known. Similar results were obtained by Zhuang et al. (2016) in experiments involving sandy soil samples. This effect can be attributed to the hysteresis in the capillary pressure–saturation relationship.

### Results of Simulations with Richards Model

We simulated this experiment using Richards model and RCM. The saturation distribution along the thickness of top layer, obtained from Richards model, was used to calculate average saturation of top layer as a function of time. Resulting curve is plotted in Fig. 5. The model predicts very fast drainage, reaching the equilibrium saturation of about 88% in no time. We see that it is far from measured data points. Note that the equilibrium value obtained from the model is actually dictated by the $P^{\mathrm{c}} ({S^{\mathrm{w}}})$ curve, which was measured separately.

By including a dynamic capillarity term in the Richards model, the simulation results improve significantly. Reasonably good agreement is reached between numerical simulation results and measured saturation when the $\tau$ value is set to 150,00 Pa s.

**Fig. 6:**
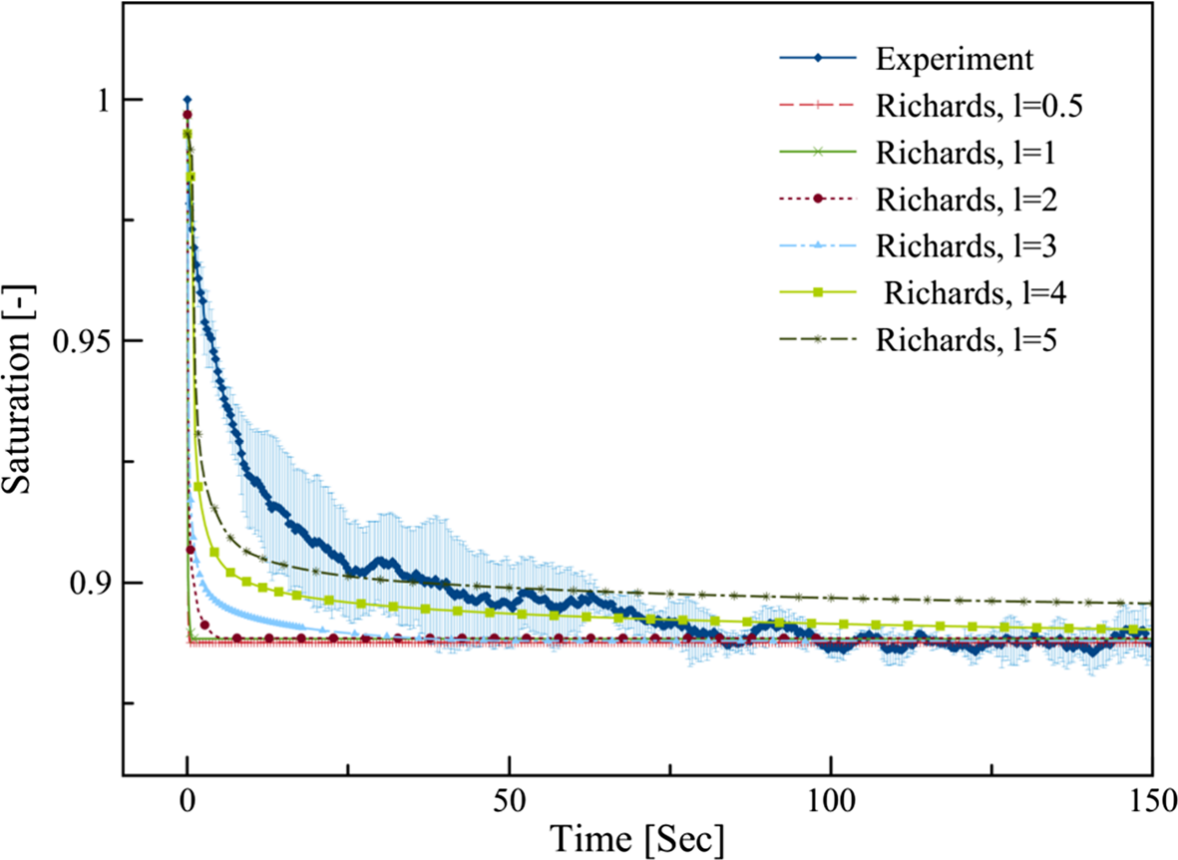
Temporal evolution of the average saturation of the top layer obtained from solving the Richards equation for different relative permeability functions and its comparison to experimental NMR data. Shaded area represents standard deviation of three replicates

**Fig. 7:**
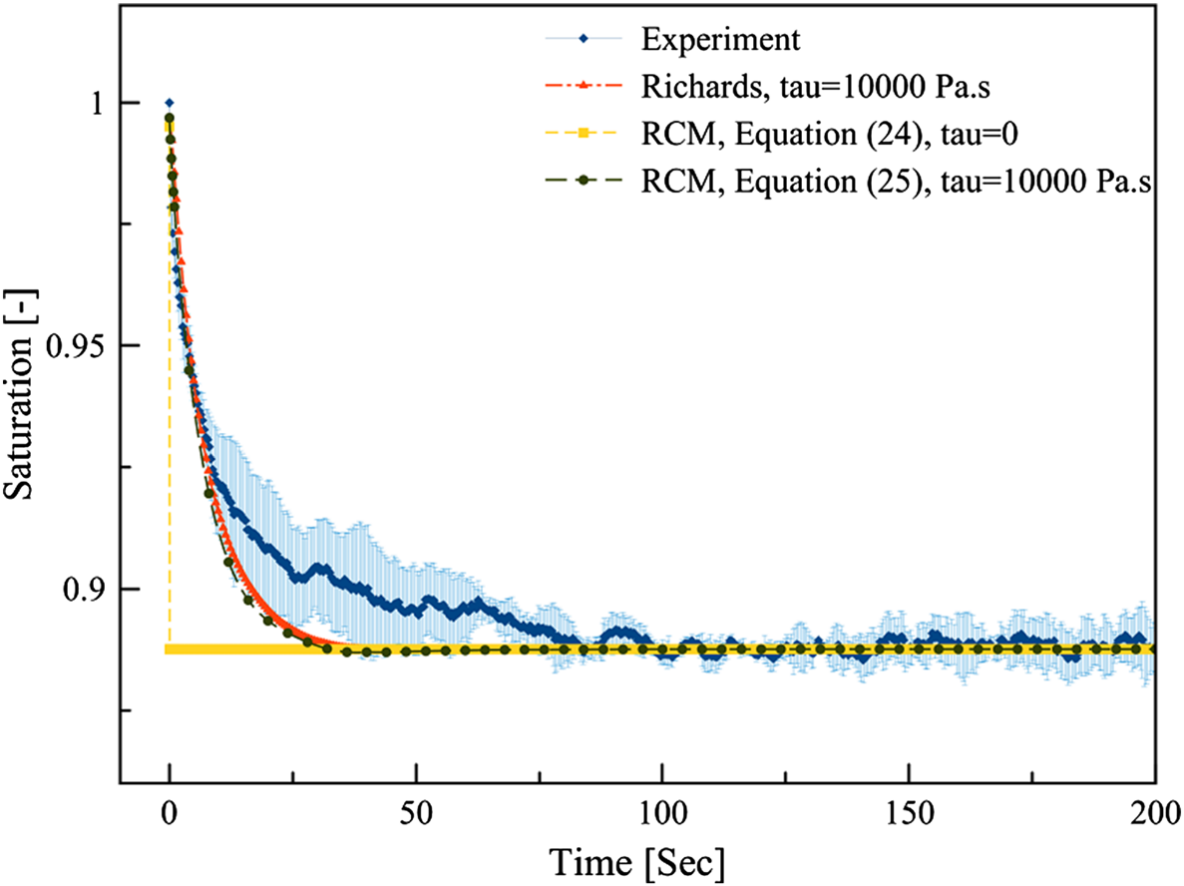
Temporal evolution of average saturation of the top layer obtained from solving the Richards equation and RCM Eqs. (24) and (25) and its comparison to experimental NMR data. Shaded area represents standard deviation of three replicates

Another approach for modifying the model results without including a dynamic effect is to reduce the relative permeability values in the very low saturation range, which is relevant to the bottom layer. We achieved this by changing the value of the coefficient *l* in Eq. (7). Although this modification slows down the rate of change of saturation (Fig. 6), it does not reproduce the experimental data satisfactorily. It should be noted that we also changed the value of the coefficient $l_{\mathrm{d}}$ in Eq. (10). However, the effect was very negligible, so that no results are shown here.

### Results of Simulations with RCM

Similar results were obtained with RCM when Eqs. (24) and (25) were solved. Results of RCM simulations, with and without dynamic capillarity term, are compared to measurements in Fig. 7. It is clear that including dynamic capillarity term improves simulation results significantly. The temporal evolution of saturation obtained from RCM is almost identical to that of Richards model in this version of RCM. However, the computational time of RCM was much smaller than that of Richards model. It should be noted that increasing the value of $\delta$ parameter used in the mass transfer coefficient $\Pi _{\mathrm{m}2}^{1,2}$, up to a value of 10, had negligible impact on final results.

**Fig. 8:**
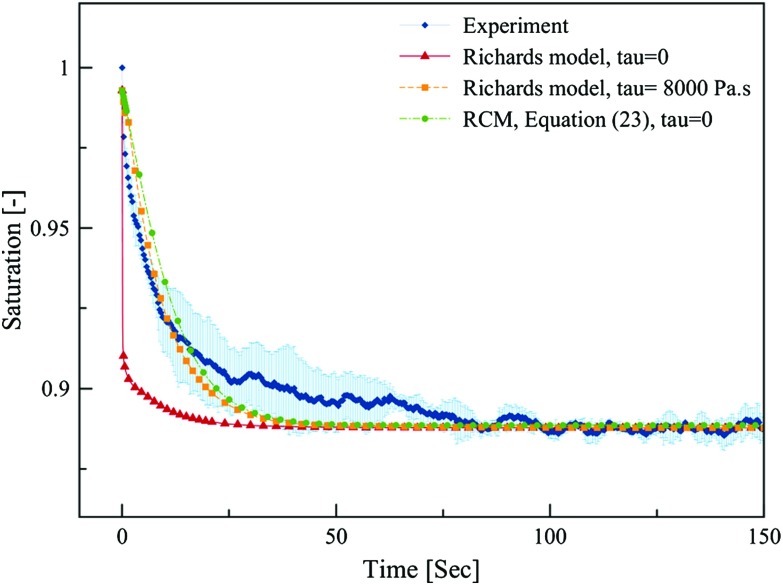
Comparison between traditional Richards model and the RCM including percolation threshold in relative permeability function in both models for the redistribution experiment. Shaded area shows standard deviation of three replicates

Next, we used the other version of RCM, Eq. (23), to simulate the change of average saturation of top layer. Results are compared to measurements data and results from the Richards model in Fig. 8. Here, we modified the relative permeability function for both models by including a percolation threshold as suggested by Landeryou et al. (2005). Therefore, a different relative permeability function was used for the saturation $S^{\mathrm{w}}$ below $S_{\mathrm{e}} =0.2$, namely $k^{\mathrm{r}} ({S^{\mathrm{w}}}) = {\upbeta } S_{\mathrm{e}}^{0.5}$, where ${\upbeta }$ is equal to the value $k^{\mathrm{r}}$ at $S_{\mathrm{e}} =0.2$ obtained from Eq. (7) with the value of the coefficient *l* set to 4. It is clear that using Eq. (21) for the mass transfer coefficient in the RCM improves the simulation results remarkably. The temporal evolution of the saturation obtained from the RCM in this scenario is identical to that of the Richards model when tau is set to 8000 Pa s (see Fig. 8). Note that it was not needed to include the dynamic effect in the RCM. Furthermore, the computational time of the RCM was one order of magnitude less than that for the Richards model. While the CPU time for the Richards model was $469 \pm 14$ s, the RCM needed a CPU time of $51 \pm 12$ s only.

## Discussion and Conclusions

We have found that when a fully saturated thin porous layer is brought into contact with a dry identical layer, very small water redistribution occurs. The small saturation change occurs relatively fast, in about 1 min, and equilibrium saturation is reached within 3 min.

Two models were used for simulating the experiment; the standard unsaturated model, based on Richards equation, and including hysteresis, and a new approach called reduced continua model (RCM). We found that the Richards model shows an unrealistically fast change of saturation. Adding a dynamic capillarity term to the Richards model helped to improve the simulation results drastically.

For the RCM, we used two different formulations for the mass transfer between the two layers. One formulation is based on harmonic mean of relative permeability functions of two layers. The second formulation is based on simple average of power functions of the two saturations. We found that simple average formulation behave similar to the Richards model; it needs the addition of a dynamic capillarity effect in order to approach the measured data reasonably well. Of course, the RCM required much less (one order of magnitude) computational time than Richards model. However, results of RCM with the harmonic mean formulation for mass transfer coefficient matched the measurement data reasonably well without including any dynamic effect.

These studies show that a delaying mechanism in the model is needed to reproduce gradual change of saturation with time. This delaying mechanism can be either provided by dynamic capillarity effect within each layer or defining appropriate mass transfer coefficient in between the layers. The physical phenomenon behind this delay mechanism can be better explained when the pore-scale experiments will be performed. A current challenge remains in performing a dynamic measurement to determine the dynamic capillarity effect in thin fibrous layers. Another challenge is to conduct experiments to better observe the effect of layer–layer exchange in both imbibition and drainage processes. Furthermore, the RCM was computationally more efficient with its computational time one order of magnitude less than that of the Richards model.
